# Leukaemia stem cells: hit or miss?

**DOI:** 10.1038/sj.bjc.6603603

**Published:** 2007-02-06

**Authors:** I Glauche, M Horn, I Roeder

**Affiliations:** 1 Institute for Medical Informatics, Statistics and Epidemiology, Medical Faculty, University of Leipzig, Haertelstr. 16/18 04107, Leipzig, Germany

**Sir**,

In their publication ‘Mathematical models of targeted cancer therapy’ Abbott and Michor (2006) emphasise the role of theoretical modelling for the understanding of cancer initiation, progression and treatment. Herein, they draw a number of general conclusions from a model of BCR-ABL1-positive chronic myeloid leukaemia (CML) under imatinib treatment that has recently been published by Michor *et al* (2005). This model relies on the existence of four subsequent compartments, through which haematopoietic cell differentiation proceeds. Chronic myeloid leukaemia development is initiated by the mutation of a single stem cell, and the expansion of the malignant (i.e. BCR-ABL1-positive) clone is assumed to be completely independent of the normal cells. Imatinib treatment, which is known to specifically affect BCR-ABL1-positive cells, is assumed to act on progenitor and differentiated cells only. In contrast, malignant stem cells are not affected and continue to expand exponentially. Abbott and Michor (2006) show that these assumptions are consistent with clinical data on BCR-ABL1 transcript levels during the first year of imatinib treatment as well as after treatment cessation.

Recent data on the long-term development of CML under imatinib monotherapy show a continuing decrease of BCR-ABL1 transcript levels even after the first year of treatment (Roeder *et al*, 2006) (Figure 1A). This long-term behaviour cannot be explained within the model of CML dynamics discussed by Abbott and Michor (2006). Owing to the contribution of CML cells from the exponentially growing malignant stem cell compartment, this model inevitably predicts a relapse of BCR-ABL1 transcript levels after about 1.5 years, even under continuing imatinib treatment and without the occurrence of resistance mutations (Figure 1B).

Within the aforementioned publication (Roeder *et al*, 2006), our group proposed an alternative explanation of the imatinib effect, which is consistent with the observed short- and long-term BCR-ABL1 levels (Figure 1C) as well as with the relapse dynamics after treatment cessation. In contrast to the model described by Abbott and Michor, we predict a selective imatinib effect on proliferating BCR-ABL1-positive cells, including stem cells, whenever they are activated into cell cycle.

In the light of the clinical long-term data, complemented by our alternative explanation of the imatinib effect, the statement by Abbott and Michor – ‘the conclusion that leukaemic stem cells cannot be depleted by imatinib can safely be drawn’ – cannot be uphold. In order to correctly describe the long-term dynamics of BCR-ABL1 transcript levels, certain modifications of the model are unavoidable. Such modifications could include a (possibly reduced) imatinib effect on malignant stem cells or a saturating growth kinetics of the malignant stem cell population.

It should be noted that although our explanation of the imatinib effect is consistent with the clinically observed long-term behaviour, it still remains a hypothesis and might not be without alternative. Particularly in comparison to the hypothesis discussed by Abbott and Michor (2006), the proposed role of the cell-cycle status of leukaemic stem cells might point to an important aspect of the imatinib effect and possibly other tyrosine kinase inhibitors. It is a particular strength of mathematical models to provide testable predictions and, therefore, to guide experimental and clinical research. However, a definite answer whether any proposed mode of imatinib action is true or not can only be given by data-based validation.

## Figures and Tables

**Figure 1:**
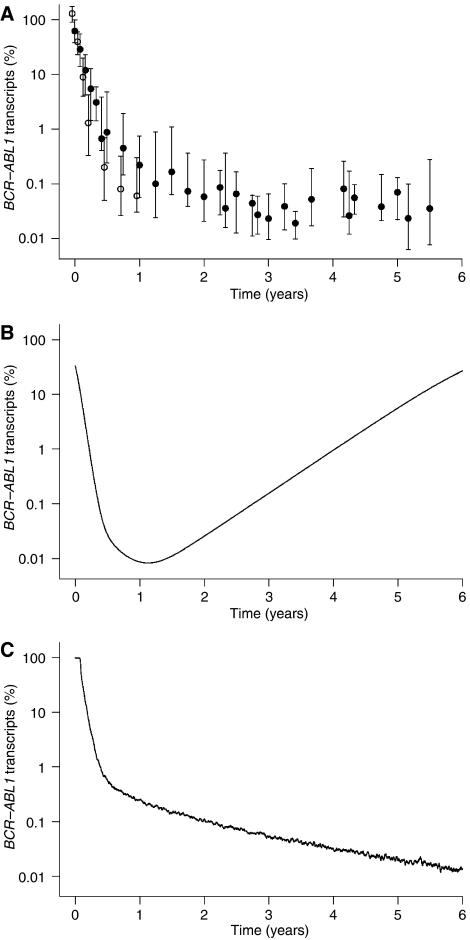
BCR-ABL1 transcript dynamics for CML under imatinib treatment: (**A**) Datapoints represent median and interquartile range of BCR-ABL1 transcript levels in peripheral blood, determined in two independent study populations: BCR-ABL1/BCR percentages of 68 individuals with imatinib-treated CML over 1 year, previously published by Michor *et al* (2005) (open circles) and BCR-ABL1/ABL1 percentages of 69 individuals with imatinib-treated CML from the German cohort of the IRIS trial over 5.5 years, previously published by Roeder *et al* (2006) (filled circles). (**B**) Long-term simulation results of BCR-ABL1 levels according to the model discussed by Abbott and Michor (2006). Parameters are taken from the original publication of this model (Michor *et al*, 2005). (**C**) Long-term simulation results of BCR-ABL1 levels according to the model introduced by Roeder *et al* (2006). Parameters are taken from the given reference.
